# Brain Phosphorus Magnetic Resonance Spectroscopy Imaging of Sleep Homeostasis and Restoration in Drug Dependence

**DOI:** 10.1100/tsw.2007.233

**Published:** 2007-11-02

**Authors:** George H. Trksak, J. Eric Jensen, Perry F. Renshaw, Scott E. Lukas

**Affiliations:** ^1^ Behavioral Pharmacology Research Laboratory, McLean Hospital, Belmont, MA, USA; ^2^ Sleep Research Program, McLean Hospital, Belmont, MA, USA; ^3^ Brain Imaging Center, McLean Hospital, Belmont, MA, USA; ^4^ Harvard Medical School, Boston, MA, USA

**Keywords:** methadone, sleep deprivation, magnetic resonance spectroscopy imaging, beta-NTP, sleep homeostasis, sleep restoration, cocaine, drug abuse, ATP, phosphorus

## Abstract

Numerous reports have documented a high occurrence of sleep difficulties in drug-dependent populations, prompting researchers to characterize sleep profiles and physiology in drug abusing populations. This mini-review examines studies indicating that drug-dependent populations exhibit alterations in sleep homeostatic and restoration processes in response to sleep deprivation. Sleep deprivation is a principal sleep research tool that results in marked physiological challenge, which provides a means to examine sleep homeostatic processes in response to extended wakefulness. A report from our laboratory demonstrated that following recovery sleep from sleep deprivation, brain high-energy phosphates particularly beta–nucleoside triphosphate (beta-NTP) are markedly increased as measured with phosphorus magnetic resonance spectroscopy (MRS). A more recent study examined the effects of sleep deprivation in opiate-dependent methadone-maintained (MM) subjects. The study demonstrated increases in brain beta-NTP following recovery sleep. Interestingly, these increases were of a markedly greater magnitude in MM subjects compared to control subjects. A similar study examined sleep deprivation in cocaine-dependent subjects demonstrating that cocaine-dependent subjects exhibit greater increases in brain beta-NTP following recovery sleep when compared to control subjects. The studies suggest that sleep deprivation in both MM subjects and cocaine-dependent subjects is characterized by greater changes in brain ATP levels than control subjects. Greater enhancements in brain ATP following recovery sleep may reflect a greater disruption to or impact of sleep deprivation in drug dependent subjects, whereby sleep restoration processes may be unable to properly regulate brain ATP and maintain brain high-energy equilibrium. These studies support the notion of a greater susceptibility to sleep loss in drug dependent populations. Additional sleep studies in drug abusing populations are needed, particularly those that examine potential differential effects of sleep deprivation.

Numerous reports have documented a high occurrence of sleep difficulties in drug-dependent populations[1,2,3], subsequently prompting sleep researchers to characterize sleep profiles and sleep physiology in drug-abusing populations. For example, poor sleep has been widely reported in cocaine-abusing populations, although studies of cocaine dependence have reported conflicting results of both hypo- and hypersomnia [4,5,6,7,8,9], which underscores the difficulty disseminating the pharmacological effects of acute drug usage on sleep from underlying changes in sleep homeostasis and abstinence. Similarly, numerous studies of alcohol, nicotine, marijuana, and opiate dependence have reported findings of poor sleep and insomnia, particularly during early abstinence[5,10,11,12,13,14,15,16,17,18]. Drug-dependent individuals actively in treatment for dependence also report various sleep difficulties. For example, sleep difficulties and insomnia particularly afflict individuals in methadone treatment[19,20,21,22,23,24]. Further investigation of sleep disturbances in drug-abusing populations is needed in order to gain understanding of how sleep-altered processes influence behavioral and cognitive performance, as well as potentially contributing to the perpetuation of drug-taking behaviors.

Sleep deprivation is a principal sleep research tool that results in marked behavioral challenges as evidenced by increased daytime sleepiness and task-specific changes in behavioral and cognitive performance[25,26,39,40]. Although sleep deprivation does not always result in impaired or altered performance, functional magnetic resonance imaging studies have identified performance-related changes in regional brain activation indicative of compensatory recruitment of functional brain regions[41,42,43]. Studies have also begun to characterize a direct role of multiple neurotransmitter systems in the regulation of sleep[32,33,34]. For example, animal studies have demonstrated increases in serotonergic activity in response to sleep deprivation in brain areas such as the dorsal raphe, hippocampus, and frontal cortex[35,36,37]. Additionally, these noted enhancements of serotonergic activity have been associated with the antidepressant responses associated with sleep deprivation reported in humans[35,36,37,38].

The resultant behavioral and physiological challenge of sleep deprivation provides a means of examining sleep homeostatic processes in response to extended wakefulness. The wakefulness presumably results in an extended period of cerebral metabolic activity and demand, allowing the examination of recovery sleep following sleep deprivation[27]. Recent research has begun to identify brain mechanisms associated with the prolonged metabolic activity of sleep deprivation as well as the enhanced restorative quality of recovery sleep[3,28,29,30,31]. Hence, the study of recovery sleep provides insight into mechanisms potentially involved in the restorative qualities of sleep. Previous magnetic resonance spectroscopy (MRS) imaging studies have examined brain markers of neurochemical abnormalities, neuronal integrity, and metabolic alterations in drug-abusing or -dependent populations[44,45,46,47,48]. For instance, studies have used phosphorus ($^{31}$P) MRS brain imaging to examine cerebral high-energy phosphate and phospholipid metabolite changes that result from long-term drug abuse and/or withdrawal in substance abusers[49,50,51]. The technique of $^{31}$P MRS imaging allows the direct measurement of brain high-energy phosphates such as alpha-, beta-, and gamma-nucleoside triphosphate (NTP)[52]. While both cytidine and guanosine triphosphates are present in the NTP resonance, the NTP resonance is derived primarily of adenosine triphosphate (ATP) and is at relatively higher concentrations (~1.8 m*M*) in the brain[53]. Beta-NTP may be of primary interest as beta-NTP is unique to NTP, whereas alpha- and gamma-NTP also contribute to nucleoside diphosphates (NDP). $^{31}$P MRS imaging provides a direct measure of NTP levels and of the currently available methods for the assessment of *in vivo* brain adenosine metabolites, $^{31}$P MRS imaging is the most commonly used[54].

A report from our laboratory previously examined how brain ATP levels fluctuate over the course of a total sleep deprivation paradigm in healthy subjects[55]. Specifically, this study demonstrated that following recovery sleep from sleep deprivation, brain high-energy phosphates, particularly beta-NTP, are markedly increased. The increase in ATP following recovery sleep may indicate a sleep homeostatic process by which the restorative processes of recovery sleep require utilization of brain ATP with the prolonged metabolic demands necessary to sustain wake-time function during sleep deprivation. These findings are consistent with animal studies indicating a reduction or depletion of brain glycogen stores[29,56,57]. Typically, the oxidation of glycogen is critical to the synthesis of ATP, which is utilized in response to metabolic demands. The neuronal utilization of brain ATP results in ATP metabolism and the by-products adenosine diphosphate (ADP) and inorganic phosphate[58,59]. Animal studies have provided strong evidence that sleep deprivation results in a build up of brain adenosine[29,42,60], which is associated with enhanced sleep pressure that results in an increased drive to sleep. Similarly, a reduction of brain glycogen stores[56], as well as a potential interaction with glucocorticoids[57], have also been demonstrated in response to sleep deprivation. Findings of brain energy changes after recovery sleep support theories that wake-time activity is sustained by brain bioenergetic restorative sleep[29,61].

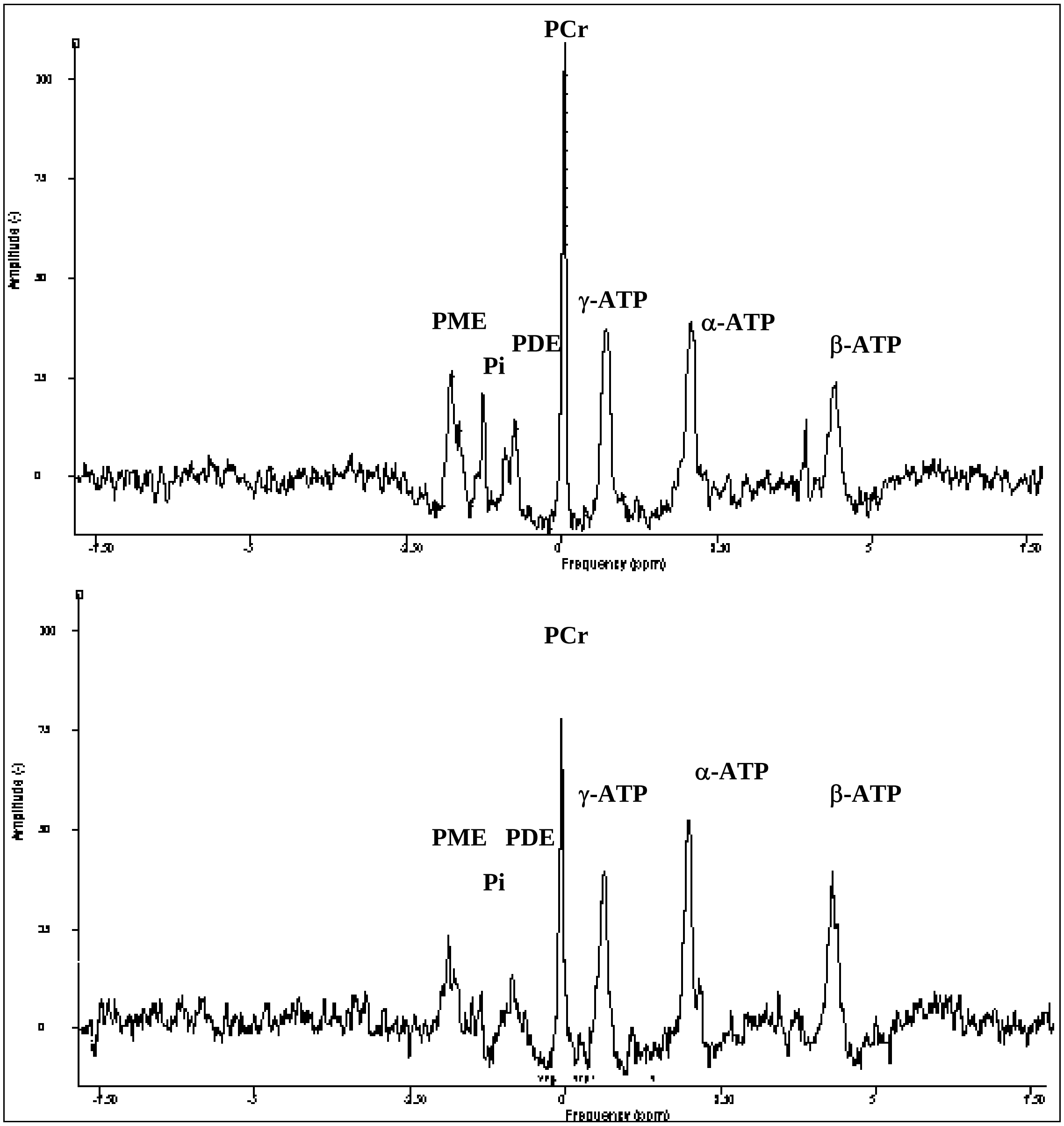

**FIGURE 1.** Illustrates global brain $^{31}P$ MRS imaging spectra indicating phosphorus-containing peaks obtained following the first night of recovery sleep in control subject (top) and MM subject (bottom).

Two more recent studies from our laboratory have begun to identify and characterize how drug-dependent populations respond to the prolonged wakefulness of total sleep deprivation, measuring both sleep physiology with polysomnography and *in vivo* measurements of brain high-energy phosphates with $^{31}P$ MRS imaging[62,63]. One study examined the response to sleep deprivation in opiate-dependent methadone-maintained (MM) subjects. Similar to the findings in control subjects, results indicated that MM subjects exhibited the greatest changes in brain ATP in the morning following recovery sleep (Fig. 1). Interestingly, MRS imaging data revealed that the enhancements in global brain beta-NTP levels over the course of the sleep deprivation paradigm were of a markedly greater magnitude in MM subjects compared to control subjects. A second study examined the effects of sleep deprivation in cocaine-dependent subjects. Most notably, cocaine-dependent subjects, similar to MM subjects, exhibited markedly greater enhancements of brain beta-NTP following recovery sleep when compared to control subjects. These preliminary data suggest that sleep deprivation in both MM subjects and cocaine-dependent subjects is characterized by underlying changes in brain ATP levels involved in sleep homeostasis and sleep restoration.

These brain MRS imaging data examining brain bioenergetics support the notion of a greater susceptibility to sleep loss in drug-dependent populations, marked by alterations in sleep homeostatic and restorative processes. Greater enhancements in brain ATP following recovery sleep may reflect a greater disruption to or impact of sleep deprivation in drug-dependent subjects, whereby sleep-restoration processes may be unable to regulate brain ATP properly and maintain brain high-energy equilibrium. A greater susceptibility to sleep deprivation may have profound functional consequences in cognitive and behavioral performance related to shift workers. Hence, additional sleep studies in drug-abusing populations are needed, particularly those that examine potential differential effects of sleep deprivation or sleep restriction. Additionally, insomnia has been associated with the occurrence of psychiatric disorders, depression, and a decline in executive function[64,65,66,67], and may have a relationship as well to decision making and the continuation of drug-taking behaviors. Greater susceptibility to sleep deprivation is also critical to the understanding of sleep processes in drug-dependent individuals in active treatment such as MM and how persistent drug abuse-related insomnia may increase relapse potential[24]. Elucidating the effects of methadone early in treatment and through the duration of therapy may be useful in extracting factors leading to failed treatment and relapse. These studies highlight the importance of developing novel clinical drug-abuse treatment approaches involving detailed monitoring and regulation of sleep behavior potentially with sleep pharmacotherapy.

## ACKNOWLEDGMENTS

The authors would like to thank the generous support provided by the National Institute of Drug Abuse; Grants DA016542, T32DA15036, DA14178, DA015116, and DA00343.

## References

1. Moreno-Coutino, A., Calderon-Ezquerro, C., and Drucker-Colin, R. (2007) Long-term changes in sleep and depressive symptoms of smokers in abstinence. *Nicotine Tob. Res.* **9(3),** 389–396.
2. Conroy, D.A. et al. (2006) Perception of sleep in recovering alcohol-dependent patients with insomnia: relationship with future drinking. *Alcohol. Clin. Exp. Res.* **30(12),** 1992–1999.
3. Johnson, M.L. et al. (2004) Modulating the homeostatic process to predict performance during chronic sleep restriction. *Aviat. Space Environ. Med.* **75(3 Suppl),** A141–146.
4. Watson, R. et al. (1992) Cocaine use and withdrawal: the effect on sleep and mood. *Am. J. Drug Alcohol Abuse* **18(1),** 21–28.
5. Pace-Schott, E.F. et al. (2005) Sleep quality deteriorates over a binge--abstinence cycle in chronic smoked cocaine users. *Psychopharmacology (Berl.)* **179(4),** 873–883.
6. Jones, E.M., Knutson, D., and Haines, D. (2003) Common problems in patients recovering from chemical dependency. *Am. Fam. Physician* **68(10),** 1971–1978.
7. Dudish-Poulsen, S. and Hatsukami, D.K. (2000) Acute abstinence effects following smoked cocaine administration in humans. *Exp. Clin. Psychopharmacol.* **8(4),** 472–482.
8. Coffey, S.F. et al. (2000) Acute and protracted cocaine abstinence in an outpatient population: a prospective study of mood, sleep and withdrawal symptoms. *Drug Alcohol Depend*. **59(3),** 277–286.
9. Johanson, C.E. et al. (1999) The effects of cocaine on mood and sleep in cocaine-dependent males. *Exp. Clin. Psychopharmacol.* **7(4),** 338–346.

10. Newcomb, M.D., Bentler, P.M., and Fahy, B. (1987) Cocaine use and psychopathology: associations among young adults. *Int. J. Addict.* **22(12),** 1167–1188.
11. Feige, B. et al. (2007) Sleep electroencephalographic spectral power after withdrawal from alcohol in alcohol-dependent patients. *Alcohol. Clin. Exp. Res.* **31(1),** 19–27.
12. Brower, K.J. et al. (2001) Insomnia, self-medication, and relapse to alcoholism. *Am. J. Psychiatry* **158(3),** 399–404.
13. Valladares, E.M. et al. (2007) EEG and vagal tone degradation during nocturnal sleep in abstinent alcohol dependence. *Sleep Med.* **8(3),** 284–285.
14. Irwin, M.R. et al. (2006) Association between nocturnal vagal tone and sleep depth, sleep quality, and fatigue in alcohol dependence. *Psychosom. Med.* 2006. **68(1),** 159–66.
15. Foster, J.H., Peters, T.J., and Kind, P. (2002) Quality of life, sleep, mood and alcohol consumption: a complex interaction. *Addict. Biol.* **7(1),** 55–65.
16. Foster, J.H. and Peters, T.J. (1999) Impaired sleep in alcohol misusers and dependent alcoholics and the impact upon outcome. *Alcohol. Clin. Exp. Res.* **23(6),** 1044–1051.
17. Byck, R. and Van Dyke, C. (1977) What are the effects of cocaine in man? *NIDA Res. Monogr.* **Series 13**, 97–117.
18. Morgan, P.T. et al. (2006) Sleep, sleep-dependent procedural learning and vigilance in chronic cocaine users: evidence for occult insomnia. *Drug Alcohol Depend.* **82(3),** 238–249.
19. Langrod, J., Lowinson, J., and Ruiz, P. (1981) Methadone treatment and physical complaints: a clinical analysis. *Int. J. Addict.* **16(5),** 947–952.
20. Oyefeso, A., Sedgwick, P., and Ghodse, H. (1997) Subjective sleep-wake parameters in treatment-seeking opiate addicts. *Drug Alcohol Depend.* **48(1),** 9–16.
21. Kay, D.C. (1975) Human sleep and EEG through a cycle of methadone dependence. *Electroencephalogr. Clin. Neurophysiol.* **38(1),** 35–43.
22. Stein, M.D. et al. (2004) Sleep disturbances among methadone maintained patients. *J. Subst. Abuse Treat.* **26(3),** 175–180.
23. Peles, E., Schreiber, S., and Adelson, M. (2006) Variables associated with perceived sleep disorders in methadone maintenance treatment (MMT) patients. *Drug Alcohol Depend.* **82(2),** 103–110.
24. Staedt, J. et al. (1996) Effects of chronic treatment with methadone and naltrexone on sleep in addicts. *Eur. Arch. Psychiatry Clin. Neurosci.* **246(6),** 305–309.
25. Pilcher, J.J. and Huffcutt, A.I. (1996) Effects of sleep deprivation on performance: a meta-analysis. *Sleep* **19(4),** 318–326.
26. Van Dongen, H.P. et al. (2003) The cumulative cost of additional wakefulness: dose-response effects on neurobehavioral functions and sleep physiology from chronic sleep restriction and total sleep deprivation. *Sleep* **26(2),** 117–126.
27. Adam, K. and Oswald, I. (1977) Sleep is for tissue restoration. *J. R. Coll. Physicians Lond.* **11(4),** 376–388.
28. Bonnet, M.H. and Arand, D.L. (2003) Insomnia, metabolic rate and sleep restoration. *J. Intern. Med.* **254(1),** 23–31.
29. Benington, J.H. and Heller, H.C. (1995) Restoration of brain energy metabolism as the function of sleep. *Prog. Neurobiol.* **45(4),** 347–360.
30. Everson, C.A., Laatsch, C.D., and Hogg, N. (2005) Antioxidant defense responses to sleep loss and sleep recovery. *Am. J. Physiol. Regul. Integr. Comp. Physiol.* **288(2),** R374–383.
31. Guseinov, G.A. (1956) [Significance of therapeutic sleep in restoration of blood quantity and morphologic composition in animals after blood loss.] *Biull. Eksp. Biol. Med.* **41(1),** 30–33.
32. Zamboni, G., Amici, R., and Perez, E. (1987) A neurochemical approach to the study of sleep. *Funct. Neurol.* **2(4),** 501–504.
33. Portas, C.M. et al. (1998) A specific role for the thalamus in mediating the interaction of attention and arousal in humans. *J. Neurosci.* **18(21),** 8979–8989.
34. Verret, L. et al. (2005) Cholinergic and noncholinergic brainstem neurons expressing Fos after paradoxical (REM) sleep deprivation and recovery. *Eur. J. Neurosci.* **21(9),** 2488–2504.
35. Gardner, J.P., Fornal, C.A., and Jacobs, B.L. (1997) Effects of sleep deprivation on serotonergic neuronal activity in the dorsal raphe nucleus of the freely moving cat. *Neuropsychopharmacology* **17(2),** 72–81.
36. Grossman, G.H. et al. (2000) Sleep deprivation stimulates serotonin release in the suprachiasmatic nucleus. *Neuroreport* **11(9),** 1929–1932.
37. Bjorvatn, B. et al. (2002) Effects of sleep deprivation on extracellular serotonin in hippocampus and frontal cortex of the rat. *Neuroscience* **113(2),** 323–330.
38. Lopez-Rodriguez, F. et al. (2003) Total sleep deprivation increases extracellular serotonin in the rat hippocampus. *Neuroscience* **121(2),** 523–530.
39. Lim, J., Choo, W.C., and Chee, M.W. (2007) Reproducibility of changes in behaviour and fMRI activation associated with sleep deprivation in a working memory task. *Sleep* **30(1),** 61–70.
40. Belenky, G. et al. (2003) Patterns of performance degradation and restoration during sleep restriction and subsequent recovery: a sleep dose-response study. *J. Sleep Res.* **12(1),** 1–12.
41. Drummond, S.P. et al. (2005) Compensatory recruitment after sleep deprivation and the relationship with performance. *Psychiatry Res.* **140(3),** 211–223.
42. Zeitzer, J.M. et al. (2006) Extracellular adenosine in the human brain during sleep and sleep deprivation: an in vivo microdialysis study. *Sleep* **29(4),** 455–461.
43. Stricker, J.L. et al. (2006) The impact of sleep deprivation and task difficulty on networks of fMRI brain response. *J. Int. Neuropsychol. Soc.* **12(5),** 591–597.
44. Chang, L. et al. (1997) Neurochemical alterations in asymptomatic abstinent cocaine users: a proton magnetic resonance spectroscopy study. *Biol. Psychiatry* **42(12),** 1105–1114.
45. Nordahl, T.E. et al. (2005) Methamphetamine users in sustained abstinence: a proton magnetic resonance spectroscopy study. *Arch. Gen. Psychiatry* **62(4),** 444–452.
46. Chang, L. et al. (1999) Cerebral (1)H MRS alterations in recreational 3, 4-methylenedioxymethamphetamine (MDMA, "ecstasy") users. *J. Magn. Reson. Imaging* **10(4),** 521–526.
47. Ende, G. et al. (2005) Monitoring the effects of chronic alcohol consumption and abstinence on brain metabolism: a longitudinal proton magnetic resonance spectroscopy study. *Biol. Psychiatry* **58(12),** 974–980.
48. Kaufman, M.J. et al. (1996) Magnetic resonance studies of substance abuse. *Semin. Clin. Neuropsychiatry* **1(1),** 61–75.
49. Silveri, M.M. et al. (2004) Cerebral phosphorus metabolite and transverse relaxation time abnormalities in heroin-dependent subjects at onset of methadone maintenance treatment. *Psychiatry Res*. **131(3),** 217–226.
50. Christensen, J.D. et al. (1996) Abnormal cerebral metabolism in polydrug abusers during early withdrawal: a 31P MR spectroscopy study. *Magn. Reson. Med.* **35(5),** 658–663.
51. Kaufman, M.J. et al. (1999) Cerebral phosphorus metabolite abnormalities in opiate-dependent polydrug abusers in methadone maintenance. *Psychiatry Res.* **90(3),** 143–152.
52. Younkin, D.P. (1993) Magnetic resonance spectroscopy in hypoxic-ischemic encephalopathy. *Clin. Invest. Med.* **16(2),** 115–121.
53. Pissarek, M. et al. (1998) Changes by short-term hypoxia in the membrane properties of pyramidal cells and the levels of purine and pyrimidine nucleotides in slices of rat neocortex; effects of agonists and antagonists of ATP-dependent potassium channels. *Naunyn Schmiedebergs Arch. Pharmacol.* **358(4),** 430–439.
54. Moore, C. and Renshaw, P. (1997) Magnetic resonance spectroscopy studies of affective disorders. In *Brain Imaging in Clinical Psychiatry*. Marcel Dekker: New York. pp. 185–214.
55. Dorsey, C.M. et al. (2003) Phosphorous31 magnetic resonance spectroscopy after total sleep deprivation in healthy adult men. *Sleep* **26(5),** 573–577.
56. Kong, J. et al. (2002) Brain glycogen decreases with increased periods of wakefulness: implications for homeostatic drive to sleep. *J. Neurosci.* **22(13),** 5581–5587.
57. Gip, P. et al. (2004) Glucocorticoids influence brain glycogen levels during sleep deprivation. *Am. J. Physiol. Regul. Integr. Comp. Physiol.* **286(6),** R1057–1062.
58. O'Neal, C.C. and Boyer, P.D. (1984) Assessment of the rate of bound substrate interconversion and of ATP acceleration of product release during catalysis by mitochondrial adenosine triphosphatase. *J. Biol. Chem.* **259(9),** 5761–5767.
59. Boyer, P.D. et al. (1982) ATP and ADP modulations of catalysis by F1 and Ca2+, Mg2+-ATPases. *Ann. N. Y. Acad. Sci.* **402**, 65–83.
60. Benington, J.H., Kodali, S.K., and Heller, H.C. (1995) Stimulation of A1 adenosine receptors mimics the electroencephalographic effects of sleep deprivation. *Brain Res.* **692(1–2),** 79–85.
61. Ticho, S.R. and Radulovacki, M. (1991) Role of adenosine in sleep and temperature regulation in the preoptic area of rats. *Pharmacol. Biochem. Behav.* **40(1),** 33–40.
62. Trksak, G.H., Jensen, J.E., Tartarini, W.B., Maywalt, M.A., Brendel, M., Kaufman, M.J., Renshaw, P.F, Dorsey, C., and Lukas, S.E. (2007) Sleep homeostasis and restorative process in cocaine users following sleep deprivation. College on Problems of Drug Dependence 2007 Meeting Abstracts. June 16–21: Quebec City, Quebec, Canada.
63. Trksak, G.H., Dorsey, C., Jensen, J.E., Tartarini, W.L., Juliano, T., Su, Z., Cuadra, B., Kaufman, M.J., Renshaw, P.F., and Lukas, S.E. (2006) Sleep homeostasis in methadone-maintained versus control subjects. College on Problems of Drug Dependence 2006 Meeting Abstracts. June 17–22: Scottsdale, Arizona.
64. Chuah, Y.M. et al. (2006) The neural basis of interindividual variability in inhibitory efficiency after sleep deprivation. *J. Neurosci.* **26(27),** 7156–7162.
65. Ford, D.E. and Kamerow, D.B. (1989) Epidemiologic study of sleep disturbances and psychiatric disorders. An opportunity for prevention? JAMA **262(11),** 1479–1484.
66. Tsuno, N., Besset, A., and Ritchie, K. (2005) Sleep and depression. *J. Clin. Psychiatry* **66(10),** 1254–1269.
67. Clark, C.P. et al. (2006) Does amygdalar perfusion correlate with antidepressant response to partial sleep deprivation in major depression? *Psychiatry Res.* **146(1),** 43–51.
